# High Operating Temperature and Low Power Consumption Boron Nitride Nanosheets Based Broadband UV Photodetector

**DOI:** 10.1038/srep42973

**Published:** 2017-03-03

**Authors:** Manuel Rivera, Rafael Velázquez, Ali Aldalbahi, Andrew F. Zhou, Peter Feng

**Affiliations:** 1 Department of Physics, University of Puerto Rico, San Juan, 00936-8377, PR/USA; 2 Department of Chemistry, King Saud University, Riyadh 11451, Saudi Arabia; 3 Department of Physics, Indiana University of Pennsylvania, Indiana, PA 15705, USA

## Abstract

We extend our work on the use of digitally controlled pulsed laser plasma deposition (PLPD) technique to synthesize high quality, 2-dimensional single crystalline boron nitride nanosheets (BNNSs) at a low substrate temperature for applications in high-performance deep UV photodetectors. The obtained sample consists of a large amount of BNNSs partially overlapping one another with random orientations. Each sheet is composed of a few (from 2 to 10) stacked atomic layers exhibiting high transparency due to its highly ordered *h*BN crystallinity. Deep UV detectors based on the obtained BNNSs were designed, fabricated, and tested. The bias and temperature effects on the photocurrent strength and the signal-to-noise ratio have been carefully characterized and discussed. A significant shift in the cut off wavelength of the BNNSs based photodetectors was observed suggesting a band gap reduction as a result of the BNNSs’ collective structure. The newly designed photodetector presented exceptional properties: a high sensitivity to weak intensities of radiation in both UVC and UVB range while remaining visible-blind, and a high signal-to-noise ratio operation even at temperatures as high as 400 °C. In addition, the BNNSs based photodetector exhibited potential for self-powered operation.

Solar-blind UV photodetectors have attracted intense attention because of a variety of potential applications in the fields of military defense, environmental monitoring and ultraviolet astronomy. During the last decade, the development of innovative UV photodetectors has experienced considerable progress^1^,^2^. This is partially attributed to newly developed techniques employed in the growth of high-quality wide-band-gap semiconductors such as crystalline gallium nitride (GaN)^3^,^4^,^5^, aluminum nitride (AlN)^6^, silicon carbide (SiC)^7^,^8^, diamond^9^,^10^, zinc oxide^11^,^12^, titanium oxide^13^ and other wide-band-gap semiconducting materials and films. These materials have been successfully applied to create various types of deep UV photodetector architectures including photoconductors, metal-semiconductor-metal (MSM) diodes, Schottky barrier and p-i-n photodiodes^14^,^15^,^16^. However, there are scarce reports of UV photodetectors capable of low-power operation and at temperatures above 150–200 °C^17^.

For this reason, Boron Nitride (BN) has recently received increased attention. BN offers several attractive features, such as chemical inertness, outstanding long term stability when operated under high-intensity UV radiation and at high operating temperatures, and tunable band gap within the UV range^18^,^19^. These properties make BN an ideal UV sensing material for particular applications such as monitoring the UV spectrum in harsh environments without the need for solar rejection filters. Several work related to cubic boron nitride (c-BN) based solar-blind UV photodetectors have been reported^20^,^21^. The main challenge with this BN structure has been the difficulty to synthesize high quality c-BN^22^. Alternatively, hexagonal BN (*h*BN) films have been proposed for the development of deep ultraviolet photonic devices^23^,^24^. As a result, a variety of *h*BN low dimensional (LD) nanostructures have been successfully synthesized including nanorods^25^, nanotubes^26^ and nanosheets (BNNSs)^27^,^28^. Among these, there is particular interest in BNNSs because they are isoelectric analogs of graphene and possess many similar physical properties and structural characteristics^29^. Many results on the synthesis of LD boron nitride materials have been reported in recent years but most rely on special CVD technique which requires extremely high temperatures (>1000 °C) and ultrahigh vacuum (UHV) for the production of BNNSs. Synthesis of BNNSs under such conditions may cause serious stresses between the substrate and the BNNSs as well as significantly increasing the cost of production^30^,^36^,^37^,^38^.

In our previous papers, we reported on the application of PLPD at low substrate temperatures for the synthesis of BNNSs^36^ and their potential use as Schottky diodes^39^, gas sensors^40^ and deep UV detectors^41^,^42^,^43^. However, during performance tests, significant atom diffusion occurred at the BNNSs-substrate interface which significantly affected the properties of the fabricated electronic device, particularly at high operating temperatures. As a result, the obtained electronic devices were not able to operate at temperatures above 200 °C^41^.

In the present work, large amounts of high-quality BNNSs have been obtained with improved BNNS-substrate properties, with which high performance deep UV detectors with large cut-off wavelength shift, high thermal resistance and low energy consumption have been fabricated. In order to improve high temperature operation capabilities, a flat buffer layer has been added on to the interface to protect sensing material from atom diffusion. The fabricated detector appears to have a very stable baseline and excellent repeatability; even when the operating temperature was up to 400 °C. The ability to operate at such high temperatures is a considerable improvement over most commercial SiC^7^, diamond^9^ or other oxide semiconductors^11^ based photodetectors that normally do not tolerate temperatures above 150 °C. It was also found that the cut-off wavelength of the BNNSs-based photodetector had shifted to longer UV wavelength, capable of detecting UVB light of up to 360 nm in wavelength. In addition, the BNNSs based photodetector exhibited potential self-powered (without external power supply) operation. This phenomenon is very different from all previous reports on BNNS UV sensing performances. The need of an external power supply in traditional deep UV photodetectors, not only increases the system’s size, cost and energy consumption, but also limits their applicability in long-term constant UV monitoring in unmanned hazardous environments. To the best of our knowledge, this is the first time a material capable of broadband UV sensing in extreme high temperature conditions while requiring almost no energy to operate is reported.

## Synthesis and characterization of BNNSs

Digitally controlled CO_2_ pulsed laser plasma deposition (PLPD) technique was used to synthesize large amounts of BNNSs at low substrate temperature. Low temperature deposition reduces the films’ stress allowing for large yields of flat BNNSs. A detailed description of the PLPD system can be found in our previous papers^36^,^44^. Briefly, a high power, pulsed (pulse width: 2 μs; repeatable rate: 5 Hz; pulse energy: 5 J) CO_2_ laser, focused with a 30 cm focal length ZnSe lens, was incident at 45 degree to a rotated (speed of *circa* 150~200 *rpm*) pyrolytic hexagonal boron nitride (BN) target (99.99% purity, B/N ratio ~1.05, density ~1.94 g/cc) inside a vacuum chamber. The laser beam focus spot on the surface of the target was about 2 mm in diameter, yielding a power density of approximately 2 × 10^8^ W/cm^2^ per pulse. Wide bandgap AlN wafers were used as substrates and placed 4 cm away from the BN target. The substrate temperature was controlled at around 350~400 °C. The duration of the deposition was 15 minutes. This process yielded hexagonal boron nitride samples with thickness up to 1.5 μm. Before deposition, 5 nm flat BN buffer layer was deposited over the AlN substrate in order to ensure high quality interface properties and protect the 2D BNNSs from atom diffusion at high temperatures. The nanoscale morphologies of BNNSs were characterized using a scanning electron microscope (SEM) and a transmission electron microscopy (TEM). Further structural and composition characterizations were performed by Raman scattering, X-ray diffraction (XRD), Fourier transform infrared spectroscopy (FTIR) and X-ray photoelectron spectroscopy (XPS). Finally, BNNSs-based deep UV photodetectors were designed, fabricated, and tested.

### Images

**Figure 1:**
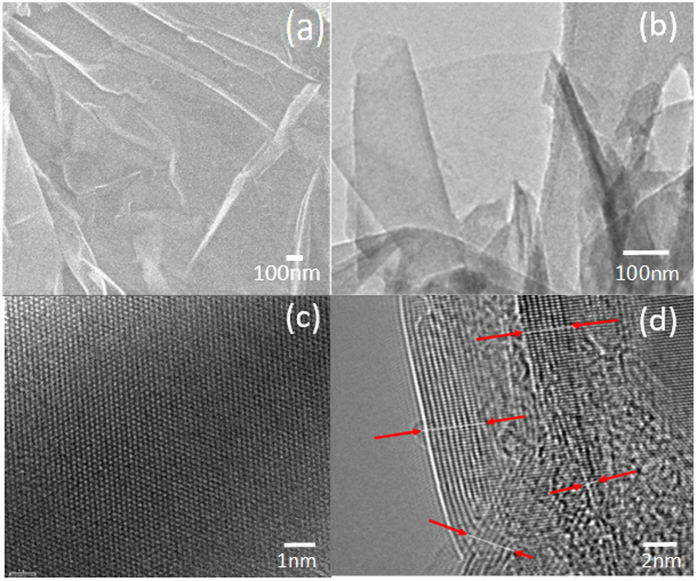
(**a**) SEM image and (**b**) TEM image of BNNSs prepared on AlN substrate. (**c**) Typical magnified HRTEM images of the selected surface area and (**d**) the edge area of the BNNSs, indicating sheet consists of a few (from 2 to 10) stacked atomic layers.

SEM studies were carried out to observe the nanoscale morphology of the nanosheets. Figure 1a shows a typical SEM image of our BNNSs prepared on AlN substrate. As seen in the figure, the sample consists of multiple rippled structures composed of BNNSs. These BNNSs clusters covered the whole surface of the 1 cm^2^ substrate. Figure 1b shows a TEM image of the BNNSs sample. In this image, the high transparency of the BNNSs is clearly visible. Slightly dark shades in the image represent overlapping or ripple areas. Results from a high resolution TEM (HRTEM), presented in Fig. 1c and d, were used to investigate the structures of center and edge areas of a BNNS. The typical honeycomb structure of *h*BN can be easily identified as well as the layer growth properties. The synthesized BN sample consists of multiple BNNSs that overlap one another with random orientations. The sheets were found to be nearly defect-free and exhibited the characteristic high-order boron and nitride atom arrays. The B-N distance was determined to be 0.14 nm, which is in good agreement with what has been reported in the literature^39^. Figure 1d provides a visual confirmation that each sheet consists of 2 to 10 stacked atomic layers, where each fringe in Fig. 1d represents a single atomic layer. The thickness of each atomic layer was determined to be 0.33 ± 0.01 nm.

The formation of the BNNSs typically relies on the selected method of fabrication, conditions and the type of substrate used. In the case of the PLPD synthesis technique applied in the present work, the heat-driven mechanical exfoliation dominates as the main process in the formation of crystalline boron nitride nanosheets. This has been confirmed by several solid pieces of evidence^36^,^45^.

### Raman Spectroscopy

**Figure 2:**
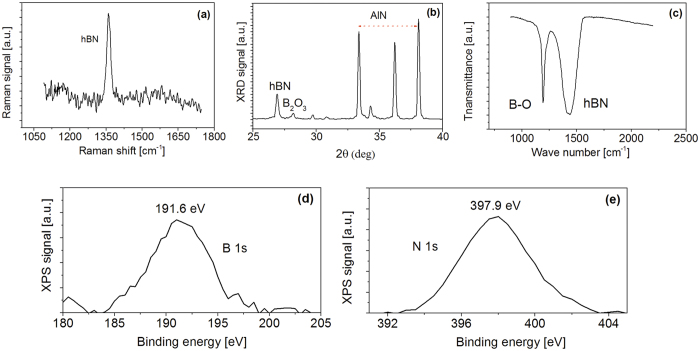
(**a**) Raman scattering spectrum, (**b**) XRD, (**c**) FTIR and (**d,e**) XPS of BNNSs prepared on AlN substrate.

Raman spectroscopy based on a triple monochromator with a 514 nm wavelength Ar^+^ ion laser was carried out to determine the crystalline structures of the BNNSs. A detailed description of the Raman scattering system employed can be found in one of our previous publications^8^. The obtained Raman data of the BNNSs is displayed in Fig. 2a. A sharp and intense Raman active E_2g_ mode at approximately 1365 cm^−1^ corresponds to *h*-BN^46^. This narrow Raman spectral profile confirms what we observed in the HRTEM measurements (Fig. 1c) which is that the PLPD technique yielded high quality BNNSs where no carbon or other impurities were detected^36^.

### XRD Results

XRD data of the sample is presented in Fig. 2b. Strong XRD peaks are observed in the range of 33–38 degrees and correspond to the AlN substrate. The peak centered at 2θ ≈ 26.9° is assigned to the *h*BN. This result is in agreement with the data obtained from Raman spectroscopy. The average spacing between the atomic layers in the BNNSs was measured to be 0.34 nm, corresponding to the (002) interplanar distance of h-BN (0.33 nm). We have reported on increased interlayer spacing of *h*BN nanosheets compared to bulk in the past^45^, and attributed to a nanoscale length effect. The increased interlayer distance in BNNSs is known to be a contributing factor in band gap width depression^41^. The thicknesses of numerous BNNS clusters were measured using HRTEM images and averaged approximately 3.0 nm, corresponding to about 9 atomic layers of *h*BN. One small peak situated at around 2θ ≈ 28° is related to B_2_O_3_ content present in the BNNSs. This contribution is possibly the result of residual gas present in the chamber or due to oxygen adsorption during the transport of the sample to characterization chambers.

### FTIR Results

Figure 2c shows the FTIR spectrum of the BNNSs. A peak centered at around 1429 cm^−1^ is associated with the in-plane E_1u_ B-N bond stretching vibration of sp^2^-bonded *h*-BN phase^46^. The actual peak position of the pure bulk *h*-BN is around 1365 cm^−1^. The observed shift in the FTIR hBN related peak towards higher wave number is possibly caused by stress induced by the subsequent B_2_O_3_ component inside the sample or lattice mismatch induced line defects propagating from the substrate. As seen from the FTIR spectrum, a relatively strong B-O associated peak at 1192 cm^−1^ was clearly detected. This is in agreement with the data obtained from the XRD measurements previously discussed.

### XPS Results

XPS is a quantitative spectroscopic technique for surface characterization. The results related to the composition of the BNNSs sample are shown in Fig. 2d and e. The sample presents the B 1s-core level peak at 191.6 eV and the N 1 s peak at 397.9 eV. Compared to XPS data from the pure bulk *h*-BN^47^, the obtained B 1s-core level in Fig. 2d appears blue shifted by 1 eV, whereas N 1s-core level in Fig. 2e has red shifted by 0.4 eV. Furthermore, the obtained profile shows slightly broad and unsymmetrical features compared with pure *h*-BN membranes. In fact, in agreement with the obtained XRD and FTIR data, both the N 1 s and B 1 s core XPS spectra of the *h*-BN membrane can be deconvoluted into a BN contribution and a corresponding oxide component.

## Results and Discussion

**Figure 3:**
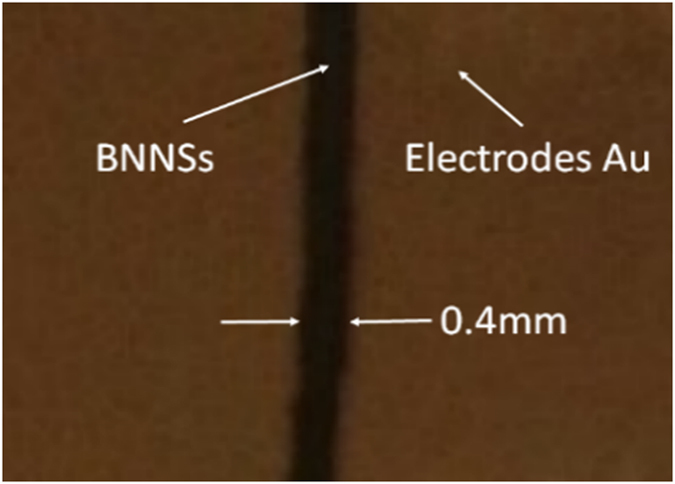
Photo image of the prototype. The Width of the gap is 0.4 mm and the length is 4 mm. The thickness of Au electrodes is 80 nm. The thickness of the boron nitride sample is around 1.5 μm and the buffer layer thickness is around 5 nm.

In order to create a testable BNNSs based photodetector prototype, plasma sputtering deposition technique (PSDT) was used for the incorporation of electrodes. As detailed in our previous reports^48^,^49^, when PSDT is used for electrode fabrication, annealing treatment has not shown to significantly improve the UV detection performance of a BNNSs based device. Therefore, in the present case 80 nm thick Au electrodes were directly deposited onto the as-grown BNNSs sample by using PSDT without any annealing before or after the electrode deposition. Compared with lithography and plasma etching, PSDT is a faster and more cost-effective fabrication method of solar blind deep UV detector prototypes. An optical image showing the current BNNSs based fabricated sensing device is presented in Fig. 3. The width of the gap between the two Au electrodes was 0.4 mm and the length of the BN-Au interphase was 4 mm. This simple electrode configuration has proved to dramatically reduce the cost and production time, allowing for rapid prototyping of high performance BNNSs based photodetectors while retaining signal quality.

### Bias Effect

**Figure 4:**
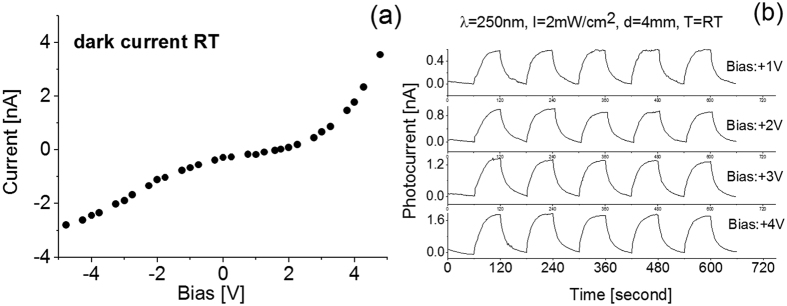
Detector current as function of bias at room temperature (RT). (**a**) Dark current; (**b**) photoresponse to 250 nm wavelength light source of 2 mW/cm^2^ power density and positioned 4 mm away from light source. T: temperature; λ: wavelength; I: light power density; d: the distance between the detector and the light lamp.

Following fabrication of the BNNSs based photo detecting device, the current–voltage (I–V) characteristics were measured using an HP34401 multimeter, an Agilent 6268B Power Supply and a Keithley 6517A electrical meter. The dark current and photocurrent measurements were carried out in standard ambient conditions. Figure 4a shows the typical current-voltage characteristics of the BNNSs in dark at room temperature. Nonlinear current-voltage curve was observed around zero applied bias, which indicates a diode behavior of the BNNS/Au contacts. The increase of current with applied bias voltage is due to an increase of the carrier drift velocity. The current measured through the BNNSs sample increases significantly under UV light illumination. Figure 4b shows the typical photocurrent response strength (I_ph_) as a function of bias when the prototype is cycled with a period of 2 minutes between the switch-on and switch-off of the 250 nm UV light source at room temperature. The differences between the UV on-off cycles can be easily identified. Higher bias yields higher response or photocurrent. The obtained photocurrent, I_ph_, was 0.5 nA, 0.9 nA, 1.3 nA and 1.7 nA at biases of +1 V, +2 V, +3 V and +4 V, respectively. The rise time was found to be slightly shorter than the recovery time, independently of the bias applied. As can be seen in Fig. 4b, the BNNSs based photodetector exhibits a clean response with high signal-to-noise ratio, independently of bias magnitude, with a well-defined and stable baseline.

**Figure 5:**
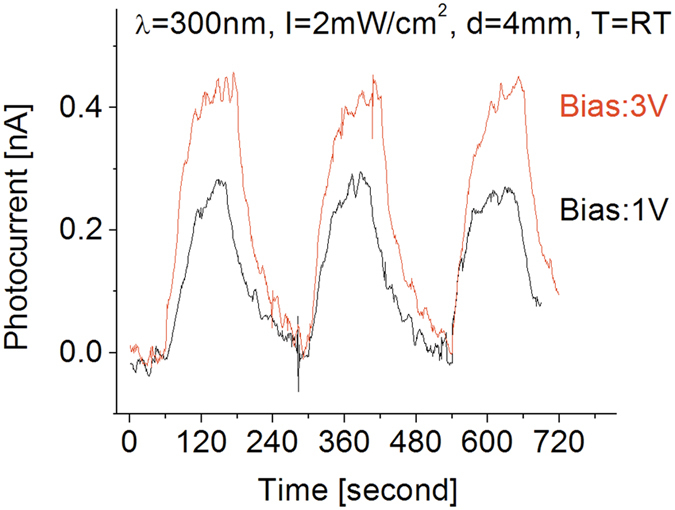
Bias effect on the response of the photodetector to 300 nm UV light.

Similar characterization was also performed with the fabricated detector exposed to 300 nm UV light illumination. Figure 5 shows the responses at room temperature when the prototype was cycled with an on-off period of 4 minutes under 300 nm UV light source at different biases. Similar to the case of the photodetector exposed to 250 nm UV light, a higher photocurrent was observed as the applied bias was increased. However, the response obtained and illustrated in Fig. 5 is considerably weaker than the one obtained under the 250 nm light source (Fig. 4). This can be directly attributed to the bandgap properties of the BNNSs under investigation.

**Figure 6:**
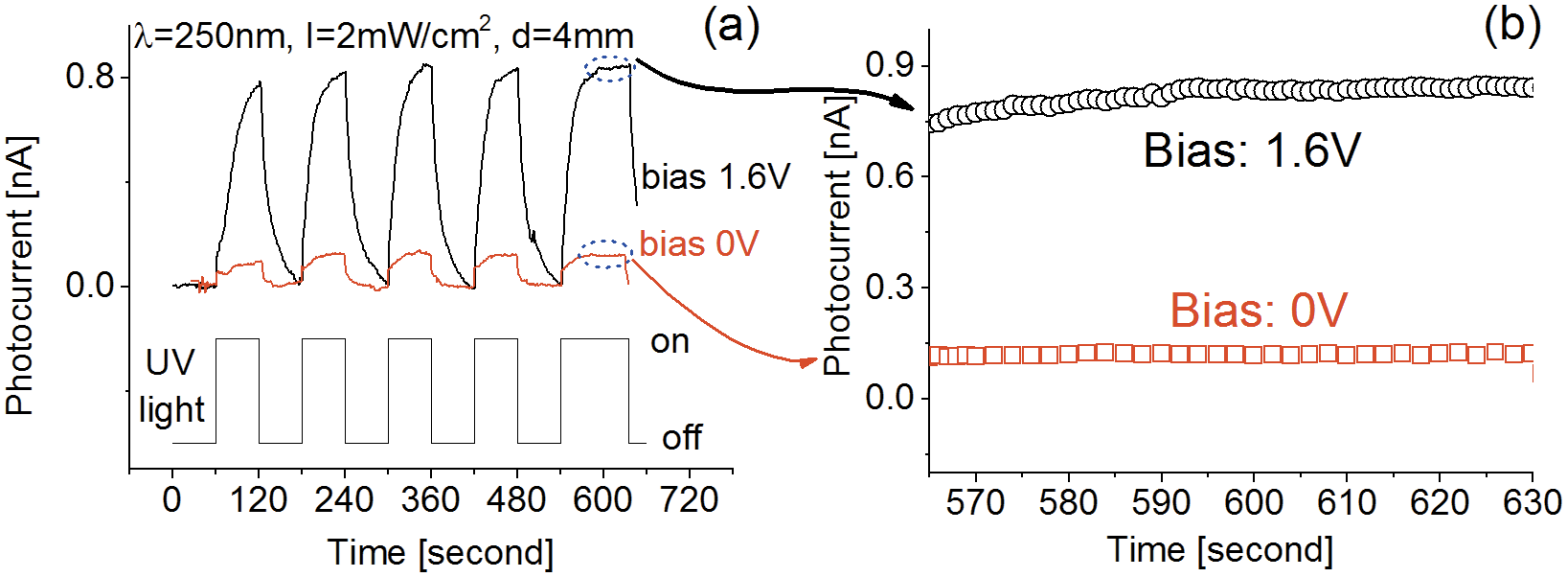
(**a**) Responses at 1.6 V bias and zero voltage when the prototype was cycled with a period of 2 minutes between the switch-on and switch-off of the 250 nm UV light source at room temperature. (**b**) Steady state photocurrent.

In order to explore the potential application as a self-powered photodetector, similar experiments as the ones to those presented in Figs 4 and 5 were carried out at zero bias. The results are presented in Fig. 6a, where, for comparison, the experimental data obtained at a bias of 1.6 V has been added. The prototype was cycled with an on-off period of 2 minutes under a 250 nm UV light source. From Fig. 6a, it is clear that the prototype generates a photoresponse even at zero bias, demonstrating that the BNNSs based photodetector is capable of photovoltaic mode operation^2^. The responsivity, R_λ_, defined as


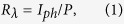


where I_ph_ is the photocurrent, P the optical power incident on the photodetector and λ the excitation wavelength, was about 9 μA/W at 0 bias and 250 nm light exposure. This value is comparable to what has been recently reported on self-powered Ga_2_O_3_ nanowire based photodetectors^50^. Besides not requiring an external energy source for operation, photovoltaic mode operation in our case appears to have the additional advantage of generating a better signal-to-noise ratio and higher stability than when a nonzero bias is applied. This is likely due to the low and stable dark current. Such improved signal-to-noise ratio and stability under zero bias can be observed in Fig. 6b, where the steady state photocurrent under biases of 1.6 V and 0 V are presented in detail. Close examination of the steady state photocurrent was possible by allowing the last cycle (250 nm UV light on) in the experiment to be 30 seconds longer.

The experimental data clearly indicated that the generated photocurrent is almost 7 times larger at 1.6 V bias than that at zero bias. However, both response time and recovery time at zero bias was found to be much shorter than that at a bias of 1.6 V. The definition of response time was based on the time duration for reaching 90% of the full response of the detector. Several works have explored and attempted to discuss the factors and mechanisms responsible for the response and recovery times of photodetectors, but most of the work has focused on ZnO based photo detectors^40^,^51^,^52^ and the subject remains fundamentally unresolved.

It was additionally noticed from Fig. 6a that at 1.6 V bias the response time of each cycle varied; the response time became shorter with the passing of each cycle. The recovery times, however, improved only slightly upon cycling. This phenomenon has been reported before in the case of SiC based deep UV photodetectors, where environmental humidity was proposed as one of the main factors influencing the performance of the detector^8^. Water molecules adsorbed on the surface would not donate electrons to the sensing materials but it would partially absorb UV light. This would affect both the response time and strength of the photodetector. The reaction between the sensing material and the water molecules would lead to a gradual formation of stable chemical bonds on the surface, causing a progressive deterioration of the response strength of the photodetector. However, when the detector is exposed to deep UV light, partial vaporization of water molecules from the detector’s surface occurs. Humidity effects on the properties of the photodetector are dependent on the competitive surface effects resulted from absorption/desorption and the related ionization/dissociation of water molecules on the photo-generated charge carriers. Subsequent heating could possibly minimize humidity effects on the performance of photodetectors. Therefore, additional experiments at different operating temperatures have been performed.

### Effects of light intensity

**Figure 7:**
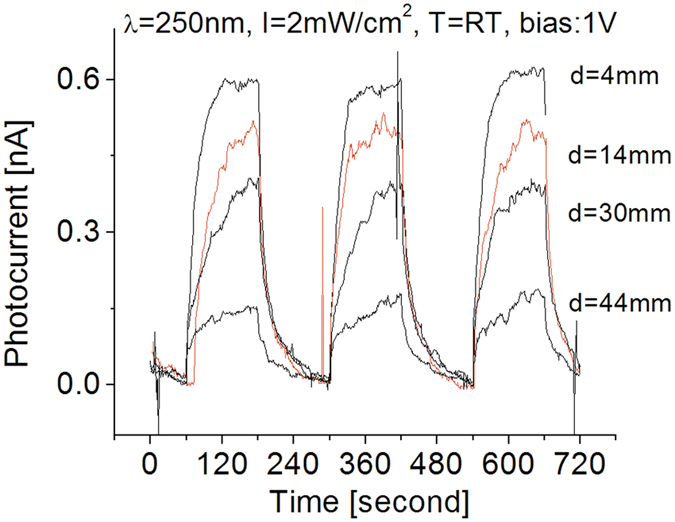
Responses at room temperature when the prototype was cycled with a period of 4 minutes between the on-off cycles of the 250 nm UV light with different intensities.

The tests with different bias voltages presented so far, indicate that high signal-to-noise ratio from the fabricated detector can be obtained at different bias levels. Therefore, a bias of 1 V has been used for the subsequent cases here presented except when specified. Figure 7 shows typical responses of the photodetector exposed to different light intensities. As in the previous measurements, when the detector was exposed to light, the induced photocurrent quickly raised and then slowly reached a relatively stable value. When the UV light was switched off, the light-induced photocurrent decreased quickly and then gradually decayed to zero. The induced photocurrent could be directly attributed to the absorption of deep UV photons. The prototype exhibited good features in repeatability and stability.

Increasing the distance between the detector and the UV light source, from 4 mm to 44 mm, the UV light intensity on the surface of the detector decreases. Since a cylinder type of UV light bulb with a diameter of 4 mm and a surface power density of 2 mW/cm^2^ was employed during characterizations, a line source model was used to estimate the intensity of the UV light irradiance on the surface of the detector. The estimated UV light intensity on the surface of the detector that was located at a distance of d = 4 mm away from the center of the bulb was around 1.0 mW/cm^2^. Similarly, we have UV light intensities of 0.28, 0.13 and 0.09 mW/cm^2^ on the surface of the detector when d = 14, 30, and 44 mm, respectively. As shown in Fig. 7, at 4 mm from the light source, the generated photocurrent from the deep UV photodetector is 0.6 nA. The obtained photocurrents almost linearly decreases as the distance between sensor and light source increases. A Similar intensity-distance relationship was observed when a 300 nm UV light source was used.

### Effective operating temperatures and cut-off wavelength

**Figure 8:**
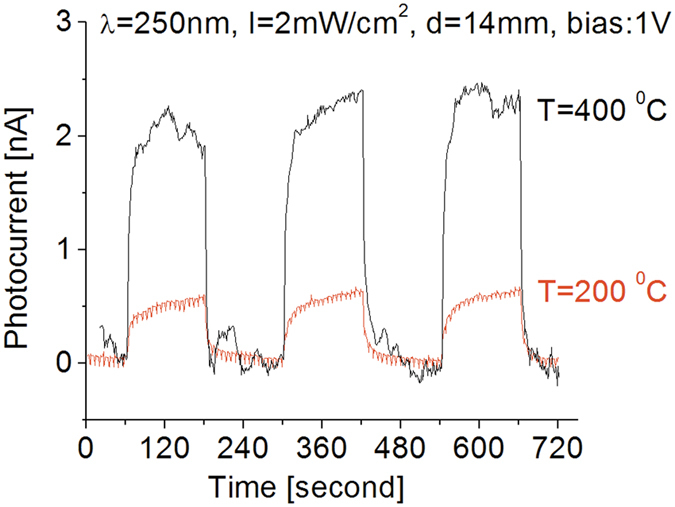
Temperature effect on the response of the detector to 250 nm UV light.

The photoresponse results of the BNNSs based photodetector under 250 nm UV illumination in the temperature range between 200 to 400 °C are shown in Fig. 8. The vast majority of effective operating temperatures reported in the past for various semiconductor based photodetectors, have consistently encountered a maximum operating temperature of approximately 200 °C or less before thermal noise completely saturates the signal of the photodetector. Comparison of the responses obtained at room temperature in Fig. 5a, and at 200 °C in Fig. 8 at 1 V bias, reveals that the obtained light-induced photocurrents remain almost unchanged within the temperature range of 25 to 200 °C. This unperturbed behavior of the BNNSs based photodetector as operating temperature increases up to 200 °C, is likely a direct result of *h*BN’s well known stability, high thermal tolerance and an effective buffer BN layer which minimizes atom diffusion at the substrate interphase even at such high operating temperatures. In an attempt to determine the prototype’s thermal operating limit, UV on-off cycling measurements were carried out at 400 °C. The results, also presented in Fig. 8, demonstrate that even when operated at 400 °C, the fabricated detector still runs with excellent features in stability and repeatability and it is likely it could perform at even higher temperatures. Such efficient operation at high temperatures is a vast performance improvement over any of our previous BNNS or SiC based photodetector prototypes^8^,^41^,^43^. In fact, besides high purity diamond, few materials have demonstrated comparable UV sensing capabilities under such high operating temperatures, making BNNSs based deep UV photodetectors ideal candidates for many scientific and industrial applications where operation of sensors at high temperatures is required^1^,^4^,^8^,^41^.

**Figure 9:**
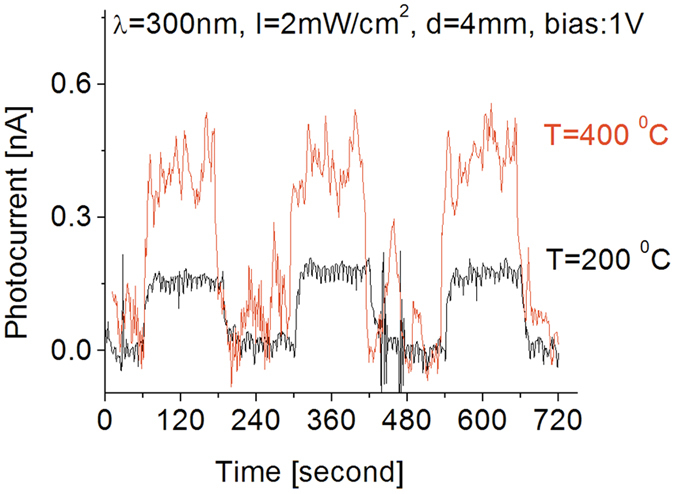
Temperature effect on the response of the detector to 300 nm UV light.

Moreover, even though the contribution of thermal noise to the photo-response of the detector was higher at higher temperatures, it was also found that increasing the operating temperature to 400 °C causes the light-induced photocurrent to increase by a factor of approximately 4 compared to the response at room temperature when exposed to 250 nm UV light. A similar phenomenon was also observed from the detector exposed to 300 nm UV light and the results are shown in Fig. 9. As seen in the figure, its response strength is much weaker than that of the detector to 250 nm UV light. No significant change in the response strength was observed following an increase in operating temperature from 25 to 200 °C, but once operating temperature reached 400 °C, 300 nm light-induced photocurrent was observed to increase almost 3 fold. Thermal noise strength, either in response strength or baseline stability, also increased by about a factor 3. As a result, the obtained signal-to-noise ratio did not change significantly.

The increase in photocurrent as the operating temperature increases has been rarely observed. Toda *et al*., observed the same photocurrent temperature dependence while investigating SiC doped with +N 53. They attributed this effect to an increase in the absorption coefficient and minority carrier diffusion length of the semiconductor. However, it is extremely challenging to determine the dominant factor.

**Figure 10:**
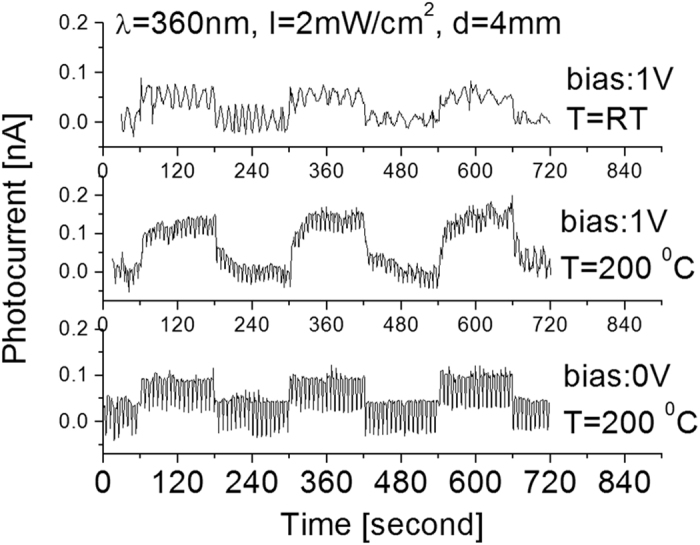
Responses when the prototype was cycled with a period of 4 minutes between the on-off cycles of the 360 nm UV light source at different applied bias and temperatures.

Experiments on the photoresponse and temperature effects of the detector exposed to 360 nm wavelength UV light were also performed in order to determine the magnitude of the cut off wavelength shift and how this particular architecture of BNNSs has affected its bandgap width for future tunable bandgap electronic devices. Figure 10 shows the responses when the prototype was cycled using a 360 nm UV light source with an on-off period of 4 minutes at different biases and temperatures. Although light-induced photocurrent is still detectable, the obtained signal is weak with poor signal-to-noise ratio.

From the HRTEM measurements of the edge area of atomic thin BNNS, it was found that the interspacing between BN atomic layers is larger than that in bulk *h*BN. The phenomenon of increased interspacing or lattice constant in the BNNSs was also confirmed by the observed peak shift in the XRD spectrum. Because the bandgap width of III-V nitride materials is generally inversely proportional to the lattice constant^54^, it is expected the decrease of the bandgap width of 2D BNNSs would unavoidably result in a red shift in cutoff wavelength. In fact, the red shift was already observed in our recent experiments with super thin BNNS based photodetectors that exhibited a sharp cut-off wavelength down to 250 nm^41^, an almost 8% shift from the cut-off wavelength of 230 nm for the bulk BN based photodetectors^47^. However, in any case, BNNSs based photodetector did not present any response to 400 nm light regardless of the temperature or the applied bias.

However, the increase in interlayer spacing of the BNNSs is not enough to account for the magnitude of the observed red shift in cut-off wavelength. The simplest explanation for addressing the prototype’s sensibility to longer wavelength UV light is the presence of impurities which contribute mid band gaps. If impurities were responsible for the apparent bandgap reduction, one would expect to observe a considerable decrease in the photocurrent and signal-to-noise ratio as the temperature increases. But in the present case the opposite was observed. As the operating temperature was increased, a stable signal-to-noise ratio and larger photoresponse was observed. Therefore, the observed shift in cut-off wavelength appears to be the result of the electronic structure of the material and not due to impurities.

Determining the electronic structure of BNNSs films is an extremely difficult task. Even in the case of bulk *h*BN, a lack of consensus within the field remains regarding its electronic structure due to the complex interplay between the interlayer positioning and the number of stacked monolayers interacting through weak Van der Waals and electrostatic forces^55^. This interlayer Van der Waals interactions is one of the main factors responsible for the difference in electronic and optical properties of few atomic layers *h*BN (BNNSs) from their bulk counterparts^56^. For example, Kang *et al*. showed via symmetry analysis that band gap and optical activity of BNNSs could be modulated by changing the number of layers as well as their interlayer orientations (stacking order)^57^.

The challenge in determining the electronic structure of the BNNSs in the current work, is compounded by additional factors, such as the presence of strains, lattice defects and layer edge effects as a result of the large number of BNNS-BNNS interphases and wide range of orientations allowed^29^,^37^,^58^,^59^,^60^. Reports on first-principles theoretical calculations have demonstrated that the band gap of few atomic layer BNNSs can be modulated by changing direction of applied electric field and by mechanical strains, effects which are likely to have an influence on the material under investigation due to the packing of randomly orientated BNNSs within the sample^45^,^61^,^62^,^63^. Additionally, theoretical and experimental results reveal that an extended line defect across an *h*BN layer can significantly reduce the bandgap^64^,^65^. Such line defect could arise from lattice mismatch at the BN-AlN interphase and would account for the peak shift observed in the FTIR data (Fig. 2c). Therefore, it is reasonable to speculate that the observed wavelength broadening of UV sensitivity in the current case is the result of a superposition of the phenomenon just described; although it is not clear the exact relationships between the thickness and orientations of super thin BNNSs material and the bandgap width related cut off wavelength shift. These questions need to be further investigated.

In conclusion, we have synthesized a micro-dimension material composed of discrete nanostructures (BNNSs). We believe that this has allowed us to take advantage of the length scale effect provided by the 2D nature of the BNNSs while retaining the attractive thermal properties of *h*BN, hence the observed ability of UV sensing at temperatures of 400 °C and possibly higher. To the best of our knowledge, we report for the first time on a potential UV photodetector capable of operating over a wide range of temperatures, up to at least 400 °C, while simultaneously having such low power requirements. These findings open a promising route into the development of effective BNNSs based UV photodetectors for long-term constant UV monitoring in unmanned hazardous environments^66^.

## Additional Information

**How to cite this article**: Rivera, M. *et al*. High Operating Temperature and Low Power Consumption Boron Nitride Nanosheets Based Broadband UV Photodetector. *Sci. Rep.* **7**, 42973; doi: 10.1038/srep42973 (2017).

**Publisher's note:** Springer Nature remains neutral with regard to jurisdictional claims in published maps and institutional affiliations.
